# Effect of income trajectory on dental service utilization patterns during the transition from childhood to adolescence: a 10-year cohort study

**DOI:** 10.1007/s00784-026-07242-7

**Published:** 2026-11-05

**Authors:** Rubia Cargnelutti, Jessica Klöckner Knorst, Bruna Brondani, Échiley da Silva Rios, Paola Mello da Silva, Thiago Machado Ardenghi

**Affiliations:** 1 Postgraduate Program in Dental Sciences, Federal University of Santa Maria, Santa Maria, Brazil; 2 Department of Stomatology, School of Dentistry, Federal University of Santa Maria, Santa, Maria Brazil; 3 Department of Pediatric Dentistry, Ribeirão Preto School of Dentistry, University of São Paulo, Ribeirão Preto, SP Brazil

**Keywords:** Child, Dental care, Longitudinal studies, Oral health, Social determinants of health, Socioeconomic status

## Abstract

**Objectives:**

This study aimed to investigate the influence of income trajectories on dental service utilization patterns during the transition from childhood to adolescence in a Southern Brazilian birth cohort.

**Materials and methods:**

Children were followed over 13 years (T1, T2, and T3). Income trajectories (stable low/decreasing vs. stable high/increasing) were identified using group-based trajectory modeling based on finite mixture models. Outcomes included frequency of dental visits and reason for the last visit (preventive vs. curative). Socioeconomic disparities were explored using the non-linear Blinder–Oaxaca decomposition method.

**Results:**

From 639 children at baseline (T1), 449 were followed at T2 and 429 at T3 (67.1% retention). Adolescents from stable high or increasing-income trajectories had a lower prevalence of dental visits for curative reasons (OR = 0.29; 95% CI 0.09–0.90). Decomposition analysis showed that maternal education (9%) and skin color (12.5%) explained only a small portion of these inequalities, suggesting an independent effect of income over time.

**Conclusions:**

Individuals with increasing income were less likely to use dental services for curative purposes, regardless of race, dental caries, and maternal education, highlighting the impact of economic stability on oral health behaviors.

**Clinical relevance:**

Income trajectories over time influence the pattern of dental service utilization in adolescence. Identifying individuals in disadvantaged socioeconomic trajectories may support targeted preventive strategies and improve access to care.

**Supplementary Information:**

The online version contains supplementary material available at https://doi.org/10.1007/s00784-026-07242-7.

## Introduction

Oral health represents a fundamental dimension of overall health and well-being, with untreated oral conditions exhibiting high global prevalence and significant negative consequences at individual, community, and societal levels [1]. These inequalities emerge from the interplay of socioeconomic determinants (particularly income, education, and healthcare access) with socially patterned behaviors, psychosocial stressors, and cumulative exposures across the life course, which together shape risk exposure and patterns of dental service utilization [2–5].

Access and utilization of dental services represent important mechanisms through which socioeconomic inequalities may influence oral health outcomes. Regular preventive dental care utilization has been associated with improved oral health status and enhanced oral health-related quality of life (OHRQoL) [2]. Service utilization is shaped by both individual and contextual factors. According to Andersen’s Behavioral Model (1995), access to health care is determined by predisposing factors (e.g., demographic characteristics and health beliefs), enabling factors (e.g., resources such as income and transportation), and perceived or evaluated needs [3]. In Brazil, previous studies have reported socioeconomic and contextual differences in dental service utilization, suggesting that income, education, and geographic vulnerability may influence access and patterns of dental care use [4–8].

Social mobility refers to changes in socioeconomic position (SEP) over time and may influence general and oral health outcomes through cumulative exposures and social pathways across the life course [9, 10]. Upward mobility has been associated with better general health indicators, including lower mortality rates and reduced chronic disease burden, as it often provides access to improved living conditions, healthcare resources, and health-promoting behaviors [10, 11]. Conversely, downward mobility has been shown to lead to worse health outcomes. In oral health, available evidence suggests that more favorable socioeconomic trajectories may be associated with lower dental caries experience, reduced tooth loss, and more favorable dental attendance patterns compared with persistently disadvantaged socioeconomic trajectories [10, 12, 13].

Although previous studies have contributed to understanding the relationship between socioeconomic status and dental attendance patterns, much of this evidence has been derived from cross-sectional designs and adult populations. However, findings from adult populations may not be directly generalizable to children and adolescents, since dental service utilization during childhood is strongly mediated by caregivers’ socioeconomic resources, knowledge, perceptions of oral health needs, and ability to access and navigate health services [14]. During the transition from childhood to adolescence, patterns of dental service utilization may be shaped by both family socioeconomic conditions and adolescents’ emerging autonomy, making this period particularly relevant for understanding how oral health care inequalities are established and maintained over time.

Longitudinal analyses of income trajectories can help clarify how socioeconomic conditions across childhood and adolescence influence dental service utilization beyond static measures of socioeconomic status. However, there is still limited evidence on how income trajectories contribute to inequalities in dental service utilization during the transition from childhood to adolescence, particularly regarding regular use and reasons for dental visits. In this sense, this study aimed to investigate the influence of income trajectories on patterns of dental service utilization during the transition from childhood to adolescence and to explore the oral health factors contributing to these inequalities.

## Methods

### Reporting and ethics procedures

The reporting of this observational study followed the Strengthening the Reporting of Observational Studies in Epidemiology (STROBE) guidelines. This study was approved by the Research Ethics Committee of the Federal University of Santa Maria (CAAE 63937422.0.0000.5346) and authorized by local and regional education authorities. Written informed consent was obtained from all guardians, and assent was also provided by the participating adolescents.

### Study design and sample

This cohort study included a 10-year follow-up period, using data collected at baseline in 2010 and at the final assessment included in this analysis in 2020. Baseline data (T1) were collected in 2010 through an epidemiological survey involving a representative sample of 639 children aged 1 to 5 years from Santa Maria, Brazil (population: 263,403; approximately 27,520 children under six). Participants were selected during the National Childhood Multivaccination Campaign. Data collection occurred at 15 of the city’s 28 primary healthcare units equipped with dental chairs, strategically located across eight administrative regions to reflect socioeconomic diversity. Within each unit, a systematic sampling method was applied, selecting every fifth child in the vaccination queue. Children with any cognitive disorder or disability were excluded from the study.

Subsequent follow-ups were conducted in four waves. This study includes data from baseline (T1), the 7-year follow-up (T2), and the 10-year follow-up (T3). All individuals from the original baseline sample (*n* = 639) were considered eligible for follow-up. Participants were traced using contact information (telephone and address) collected at baseline. Additional tracking strategies included searches via social media and visits to schools. Additional information on previous waves and related analyses from this cohort has been reported elsewhere [15–17].

The sample size calculation for the baseline study has been published previously [18]. A post hoc power analysis was conducted considering the observed odds ratio (OR = 0.29) for the association between income trajectory and the use of dental services for curative purposes. The study included approximately 610 participants in the stable high- or increasing-income group and 29 participants in the stable low- or decreasing-income group. Assuming a conventional significance level of α = 0.05 (two-tailed), the statistical power was estimated at approximately 95% to detect the observed association.

### Data collection and variables

Data of demographic, socioeconomic, and behavioral variables were collected using structured questionnaires administered through face-to-face interviews. Clinical oral examinations were conducted by trained and calibrated examiners, following the methodology recommended by the World Health Organization’s manual for oral health surveys [18]. Inter- and intra-examiner reliability for the clinical variable dental caries was assessed using Kappa statistics, with values ranging from 0.70 to 0.88 and 0.70 to 0.81, respectively.

Sociodemographic variables included sex (male or female), age (in years), and self-reported skin color, all assessed at baseline (T1). Skin color classification followed the criteria of the Brazilian Institute of Geography and Statistics and was dichotomized as White versus Black/Brown/Yellow [19]. Maternal education was measured by years of formal schooling, categorized as < 8 years or ≥ 8 years, corresponding to incomplete versus complete elementary education in Brazil.

Family income was assessed at T1, T2 and T3, based on total household income in the month prior to data collection, including wages, pensions, and government benefits. Income was recorded in Brazilian reais (BRL), with average exchange rates of approximately 1.76 BRL, 3.19 BRL, and 5.15 BRL per 1 USD in 2010 (T1), 2017 (T2), and 2020 (T3), respectively. Income trajectory was categorized into two groups across the three waves: (0) stable low or decreasing income; and (1) stable high or increasing income.

Dental caries at T1, T2 and T3 was assessed using the International Caries Detection and Assessment System (ICDAS) criteria [16, 20]. For data analysis, only cavitated carious lesions (ICDAS scores 3, 5, and 6) were considered, corresponding to the decayed component of the decayed, missing, and filled teeth index (DMFT) [18].

Income trajectory was treated as the main exposure. The outcomes were regular dental service utilization and reason for the last dental visit, both assessed at T3. Sex, skin color, maternal education, and baseline dental caries were included as covariates in the adjusted regression models (Supplementary Fig. 1). Use of dental services was measured with the question: “In the past 12 months, how many times have you visited a dentist?” Response options ranged from (0) none to (3) three times. For analysis, dental service use was categorized as regular (≥ 1 visit per year) or non-regular (< 1 visit per year), in line with WHO recommendations [18]. The reason for the last dental visit was assessed with the question: “What was the reason for your last dental visit?”, with response options including: (0) toothache; (1) other mouth pain; (2) accident or trauma; (3) routine check-up; and (4) orthodontic treatment. Reasons were dichotomized as routine (3 or 4) versus non-routine (0, 1, or 2).

### Conceptual framework and covariate selection

A conceptual framework was developed to guide the selection of covariates included in the adjusted analyses. A Directed Acyclic Graph (DAG) (Supplementary Fig. 1) was used to represent the assumed causal relationships among income trajectory, dental service utilization, and potential confounding or adjustment variables. The DAG was constructed based on theoretical frameworks addressing oral health inequalities and social determinants of health, including materialist, psychosocial, behavioral, and life-course perspectives [21–24], as well as models of health service utilization, particularly Andersen’s Behavioral Model [3] and other theoretical models of health service utilization [25]. Evidence from previous studies on socioeconomic determinants of oral health, dental caries, caregiver-mediated access to dental care, and dental service utilization in children and adolescents was also considered [2, 5, 13–17, 26, 27].

Maternal education and skin color were considered upstream social determinants that may influence both income trajectory and dental service utilization. Sex and age were considered predisposing characteristics associated with dental service utilization, and baseline dental caries was considered a need-related factor that may influence subsequent dental service utilization.

### Data analysis

Data were analyzed using Stata version 19. Descriptive statistics were used to summarize demographic, socioeconomic, behavioral, and clinical characteristics. Comparisons between participants retained in the study and those lost to follow-up were conducted using the Chi-square test for categorical variables and the t-test for continuous variables.

Income trajectories were identified using group-based trajectory modeling (Finite Mixture Models, “traj” command), a method that identifies latent subgroups of individuals following similar patterns of change in a variable over time, rather than assuming a single average trajectory for the whole sample. The number and shape of trajectory groups were selected based on model fit statistics (BIC) and the probability of group membership. This approach allowed income to be characterized as a dynamic process across the three waves, rather than as a static exposure measured at a single time point. These trajectories were then used as exposure variables in adjusted logistic regression models to examine associations with dental service utilization at T3, considering both frequency of use and reason for the last dental visit. The adjusted models included the covariates described above, selected a priori based on the conceptual framework represented by the DAG and supported by previous literature [19, 20, 25]. Results are presented as odds ratios (ORs) with 95% confidence intervals (CIs).

To investigate socioeconomic disparities in the considered outcomes, we used a non-linear extension of the Blinder-Oaxaca decomposition method, appropriate for binary outcomes. This method decomposes the observed difference in outcome prevalence between income trajectory groups into two components: an “explained” component, attributable to differences in the distribution of observed covariates (maternal education and skin color) between groups, and an “unexplained” component, attributable to differences in the effect (coefficients) of these covariates between groups, which may reflect unmeasured structural or contextual factors. The low-income trajectory group was used as the reference. This approach allowed us to quantify how much of the observed disparity could hypothetically be reduced if both income trajectory groups had the same distribution of maternal education and skin color. Both absolute and relative contributions to the total disparity (gap) were reported for each explanatory variable.

Throughout the manuscript, the term “dental service utilization” was used to refer to the use of dental care. The term “socioeconomic inequalities” was used to describe differences in outcomes across income trajectory groups, while “gap” was used specifically in the decomposition analysis to refer to the difference in outcome prevalence between groups.

## Results

Table 1 displays the sociodemographic characteristics of the study population across all waves. The cohort comprised 639 children at baseline (T1, 2010), with 449 participants remaining at the 7-year follow-up (T2, 2017) and 429 at the 10-year follow-up (T3, 2020), corresponding to a 67.1% retention rate over the 10-year study period. At baseline (T1), the sample consisted of 322 male participants (50.4%) and 502 White participants (78.5%). Most mothers had completed 8 or more years of schooling (*n* = 357; 56.5%), and 231 children presented cavitated carious lesions (36.1%). No significant differences were found in these baseline characteristics between participants retained in the study and those lost to follow-up. Income trajectory analysis revealed that 610 participants followed a stable high- or increasing-income pattern (95.5%), whereas 29 followed a stable low- or decreasing-income pattern (4.5%). By the final follow-up (T3), 249 adolescents reported regular dental service utilization (58.2%), and 215 indicated that their most recent dental visit was for routine reasons (50.6%).

**Table 1:** General characteristics of the sample of individuals evaluated at baseline (T1) and follow-ups (T2 and T3), Santa Maria, RS Brazil

Variables		
Baseline (T1)	*N*^†^ (%)	*p*-value*
Sex Male Female	332 (50.4) 317 (49.6)	0.227
Skin color White Black-brown-yellow	501 (78.5) 137 (21.5)	0.158
Maternal education *≥* 8 years < 8 years	357 (56.5) 275 (43.5)	0.669
Dental caries Absent Present	408 (63.8) 231 (36.1)	0.737
*Follow-ups*		
Income trajectory (T1, T2 e T3) Stable low-decreasing income Stable high-increasing income	610 (95.5) 29 (4.5)	0.536
Regular use of dental services (T3) Yes Not	249 (58.2) 179 (41.8)	-
Reason for using dental services (T3) Routine Non-routine	215 (50.6) 210 (49.4)	-

Table 2 presents the adjusted associations between income trajectories and dental service utilization outcomes among participants with available data for each outcome and covariates included in the regression analyses. The analytical sample comprised 428 participants for regular dental service utilization and 425 participants for reason for dental service utilization. These numbers differ from the total number of participants assessed at T3 (*n* = 429) due to missing responses to specific outcome questions. Regarding regular use of dental services, adolescents with a stable high- or increasing-income trajectory had lower odds of non-regular dental visits compared to those with a stable low- or decreasing-income trajectory, although this association was not statistically significant (OR 0.58; 95%CI 0.19–1.55). Lower maternal education was significantly associated with non-regular dental service use (OR 1.78; 95%CI 1.18–2.69). Considering the reason for dental service use, adolescents with a stable high or increasing income were less likely to report non-routine reasons for their dental visits compared to those with a stable low or decreasing income (OR 0.29; 95%CI 0.09–0.90). Additionally, being female, having a mother with less than 8 years of education, and presenting dental caries were all significantly associated with non-routine dental visits.

**Table 2:** Adjusted associations between income trajectories and variables regarding the dental service utilization

Variables	*Regular dental service utilization (n = 428)*		*Reason for dental service utilization (n = 425)*		
	Adjusted OR (IC 95%)	*p*-value		Adjusted OR (IC 95%)	*p*-value
Main exposure					
Income trajectory Stable low-decreasing income Stable high-increasing income	1 0.58 (0.19–1.55)	0.258	1 0.29 (0.09–0.90)		< 0.05
*Adjusting variables*					
Sex Male Female	1 0.77 (0.52–1.16)	0.220	1 0.63 (0.42–0.95)		< 0.05
Skin color White Black-brown-yellow	1 1.17 (0.73–1.88)	0.505	1 1.22 (0.75–2.00)		0.40
Maternal education *≥* 8 years < 8 years	1 1.78 (1.18–2.69)	< 0.001	1 1.60 (1.05–2.44)		< 0.05
Dental caries Absent Present	1 1.42 (0.94–2.14)	0.095	1 2.30 (1.51–3.52)		< 0.05

Table 3 presents the decomposition of the effect of income trajectory on regular dental service utilization among participants included in the decomposition analysis (*n* = 428). The model explains approximately 18% of the difference in the prevalence of irregular dental service use between income groups. Of this gap, 28% (0.05/0.18) is attributable to observed variables included in the model. This indicates that if individuals with a disadvantaged income trajectory had the same maternal education and skin color as those with an advantaged trajectory, the disparity would be reduced by 28%. The remaining 72% (0.13/0.18) is attributed to unobserved or unmeasured factors. Among the observed contributors, maternal education accounts for 27.7% of the gap, while skin color explains 2.2%.

**Table 3:** Effect decomposition analysis according to income trajectory and *regular dental service utilization* - Blinder-Oaxaca Model

*Regular dental service utilization (n = 428)*	Coefficient (IC 95%)^†^	Percentage
Prevalence estimates in the low-income trajectory group	0.42 (0.37-0,47)	
Prevalence estimates in the high-income trajectory group	0.23 (0.05–0.42)	
Difference	0.18 (0.00–0.37)	
*Difference due to model variables*	0.05 (0.01–0.09)	28.0%
*Differences not explained by the model*	0.13 (−0.06-0.32)	72.0%
Detailed analysis of the part explained by the model		
Maternal education	0.053 (0.01–0.09)	27.7%
Skin color	0.004 (0.00–0.01)	2.2%

Table 4 presents the decomposition of the effect of income trajectory on the reason for dental service utilization among participants included in the decomposition analysis (*n* = 425). The model explains a 32% difference in the prevalence of curative dental service use between income groups. Of this gap, 16% (0.05/0.32) is attributable to observed variables in the model. This suggests that if individuals with a disadvantaged income trajectory had the same maternal education and skin color as those with a more favorable trajectory, the gap would be reduced by 16%. The remaining 84% (0.27/0.32) is due to unobserved or unmeasured factors. Among the explained components, maternal education accounts for 9.4% of the gap and skin color for 12.5%.

**Table 4:** Effect decomposition analysis according to income trajectory and *reason for dental service utilization* - Blinder-Oaxaca Model

*Reason for dental service utilization (n= 425)*	Coefficient (IC 95%)^†^	Percentage
Prevalence estimates in the low-income trajectory group	0.51 (0.46–0.56)	
Prevalence estimates in the high-income trajectory group	0.19 (0.02–0.36)	
Difference	0.32 (0.15–0.50)	
*Difference due to model variables*	0.05 (0.01–0.09)	16.0%
*Differences not explained by the model*	0.27 (0.08–0.45)	84.0%
Detailed analysis of the part explained by the model		
Maternal education	0.03 (−0.01-0.07)	9.4%
Skin color	0.04 (−0.01-0.08)	12.5%

## Discussion

### Main findings: income trajectory and dental service utilization

This study examined the association between income trajectories and dental service utilization patterns during the transition from childhood to adolescence in a southern Brazilian cohort, while identifying factors contributing to socioeconomic inequalities in oral healthcare access. The main finding was that adolescents with stable high or increasing income were less likely to report dental visits for curative or non-routine reasons when compared with those with stable low or decreasing income. In contrast, income trajectory was not statistically associated with regular dental service utilization. These findings are consistent with life-course approaches suggesting that socioeconomic conditions experienced during childhood may shape health-related behaviors, access to services, and oral health outcomes over time [9, 10, 21, 24].

The lower likelihood of non-routine dental visits among adolescents from more favorable income trajectories may reflect differences in material resources, continuity of care, caregivers’ ability to recognize and respond to oral health needs, and opportunities for preventive-oriented dental care. From materialist and life-course perspectives, socioeconomic advantage may provide access to resources that support healthier environments, timely care, and preventive routines, whereas socioeconomic disadvantage may increase cumulative exposure to oral disease risk and barriers to care [9, 21, 23, 24]. In Brazil, previous studies have also shown that socioeconomic and contextual conditions are related to dental service utilization and oral health outcomes, although much of this evidence comes from adult or cross-sectional studies and should be interpreted cautiously when applied to children and adolescents [4–8].

Although regular dental service utilization was not associated with income trajectories, this finding should be interpreted with caution. The need for regular dental visits may vary according to individual caries risk, oral health status, perceived needs, and clinical recommendations. Therefore, the frequency of dental service utilization does not necessarily reflect access to preventive care alone, as it may also capture underlying treatment needs or recurrent oral health problems. Therefore, the lack of an association between income trajectory and regular dental service utilization does not necessarily indicate the absence of socioeconomic inequalities in dental care. Rather, this outcome may combine different patterns of utilization, including preventive attendance among adolescents with better access and problem-oriented attendance among those with greater oral health needs [2, 3, 18].

According to Andersen’s Behavioral Model, healthcare utilization is determined not only by material resources but also by predisposing, enabling, and need-related factors [3, 25]. In childhood and adolescence, these dimensions are also shaped by family and caregiver characteristics, including socioeconomic resources, perceptions of oral health needs, health literacy, and decisions regarding when and why dental care is sought [2, 5, 28, 29]. Thus, families following distinct income trajectories may report similar frequencies of dental visits, but for different reasons. This reinforces the importance of interpreting regular dental service utilization together with the reason for the last dental visit, which may better distinguish preventive from problem-oriented patterns of care.

### Decomposition findings: sources of socioeconomic inequality

The decomposition analysis helped to characterize the observed socioeconomic inequalities in dental service utilization. Maternal education contributed to part of the gap between income trajectory groups, suggesting that caregiver-related socioeconomic resources may help explain differences in patterns of dental care. However, this contribution should not be interpreted as an isolated causal effect of maternal education. Rather, maternal education may represent broader dimensions of family socioeconomic position, including access to health information, ability to understand oral health needs, capacity to navigate services, and establishment of preventive routines during childhood and adolescence [28–30]. Previous studies have shown that parental education and family socioeconomic conditions are associated with children’s oral health, dental caries, and dental service utilization, supporting the relevance of caregiver-related factors in this developmental period [2, 5, 28, 29].

Skin color also contributed to the gap, suggesting that racialized social processes may be involved in the distribution of preventive and curative patterns of care. In Brazil, skin color may operate as a marker of structural inequalities related to access to resources, exposure to discrimination, experiences within healthcare systems, and opportunities for timely preventive care [9, 26, 31, 32]. However, as with maternal education, this contribution should be interpreted within the decomposition framework and not as an isolated causal effect of skin color. These findings reinforce the need to consider broader social and institutional mechanisms when examining socioeconomic inequalities in dental service utilization [9, 21, 32].

A substantial proportion of the observed gaps remained unexplained, indicating that other factors not measured in this study may contribute to inequalities in dental service utilization. These factors may include geographic accessibility, availability and quality of public dental services, continuity of care, cultural norms regarding dental attendance, previous experiences with dental services, stigma, and other institutional or contextual barriers. This finding is consistent with theoretical approaches that conceptualize oral health inequalities as the result of multiple, interacting material, psychosocial, behavioral, and life-course pathways [21–24, 32]. Therefore, the results should be interpreted as evidence of complex pathways linking socioeconomic trajectories to dental service utilization, rather than as the effect of any single sociodemographic factor.

### Strengths, limitations and conclusion

These findings should be interpreted considering some limitations. First, the categorization of income trajectories into only two groups may have oversimplified the diversity of socioeconomic experiences over time. However, this classification was guided by the distribution of the data and allowed the identification of broader patterns of income trajectory across the follow-up. Second, although covariates were selected a priori based on a conceptual framework, residual confounding due to unmeasured individual, family, contextual, or service-related factors cannot be ruled out. It should also be noted that the adjusted models were designed to estimate the effect of income trajectory on dental service utilization; the odds ratios reported for the remaining covariates (e.g., maternal education, skin color) should not be interpreted as independent causal effect estimates, as these variables were not the primary focus of confounding control and may be subject to their own distinct sources of bias. Third, dental service utilization was based on self-reported information, which may be subject to recall bias or social desirability bias. Finally, the interpretation of regular dental service utilization is complex, since frequency of visits may reflect both access to preventive care and underlying treatment needs.

Despite these limitations, this study has important strengths. Its population-based cohort design and 10-year follow-up allowed income trajectories to be assessed from early childhood to adolescence, a key life-course period marked by increasing autonomy in healthcare use [10, 24, 27]. Investigating this period offers an opportunity to identify early determinants of oral health inequalities and to guide preventive strategies with potential long-term impact. In addition, the study considered different dimensions of dental service utilization, including both regularity of utilization and reason for the last dental visit, allowing a more nuanced interpretation of socioeconomic inequalities in oral healthcare.

In conclusion, income trajectory was associated with patterns of dental service utilization during the transition from childhood to adolescence. Adolescents with stable high or increasing income were less likely to report dental visits for curative or non-routine reasons, suggesting more favorable patterns of dental care. However, regular dental service utilization alone should be interpreted cautiously, as it may reflect both access to care and underlying treatment needs. A substantial portion of the observed inequalities remained unexplained, highlighting the complexity of the pathways linking socioeconomic conditions to dental service utilization. These findings support the need for long-term intersectoral public policies aimed at reducing socioeconomic inequalities from early childhood and promoting equitable access to preventive oral healthcare.

## Supplementary Information

Below is the link to the electronic supplementary material.

Supplementary Material 1

## Data Availability

The data that support the findings of this study are available on request from the corresponding author. The data is not publicly available due to privacy or ethical restrictions.
