# Supplementary material for: Effect of income trajectory on dental service utilization patterns during the transition from childhood to adolescence: a 10-year cohort study
Source: Clin Oral Investig. 2026 Nov 5;30(11):555. doi: 10.1007/s00784-026-07242-7 (PMC13736408; doi:10.1007/s00784-026-07242-7)
Supplement: Supplementary file 1 [file 784_2026_7242_MOESM1_ESM.docx]

Supplementary Figure 1. Directed Acyclic Graph representing the assumed causal relationships among income trajectory, dental service utilization, and covariates considered in the analytical framework. Income trajectory was defined as the exposure, and dental service utilization at T3 was defined as the outcome. Variables included in the adjusted models were sex, skin color, maternal education, and baseline dental caries.
